# A Case of Frosted Branch Angiitis in the Age of Polymerase Chain Reaction: Diagnostic and Therapeutic Considerations

**DOI:** 10.7759/cureus.28816

**Published:** 2022-09-05

**Authors:** Moises Enghelberg, David I Sierpina

**Affiliations:** 1 Retina Service, Loma Linda University Eye Institute, Loma Linda, USA; 2 Ophthalmology, Loma Linda University Eye Institute, Loma Linda, USA

**Keywords:** retina, pcr multiplex, real-time pcr and nucleic acid sequencing, uveitis, frosted branch angiitis

## Abstract

To describe a unique case highlighting the limitations and caveats of multiplex polymerase chain reaction (mPCR) in the diagnosis of posterior infectious uveitis, specifically frosted branch angiitis (FBA), we present a case of FBA in which multiple diagnostic modalities, including mPCR, are inconclusive. A thorough literature review was carried out to discuss the validity of mPCR in the setting of posterior infectious uveitis, the theoretical effect of sample dilution, and to explore a management strategy in these difficult cases. It is known that mPCR has high sensitivity and specificity, with a low false negative rate. However, the rate of false negatives appears to increase in cases of FBA, and when samples are diluted. In such cases we suggest empiric treatment be initiated and targeted towards microorganisms most likely to be implicated based on exam and history.

## Introduction

Frosted branch angiitis (FBA), is an important posterior uveitic condition that was first clinically recognized in 1976 by Ito and co-workers who described a severe inflammatory syndrome consisting of vasculitis, retinitis, and vitritis in a 6-year-old child post-vaccination [1]. The striking clinical appearance in which inflammatory perivascular deposits aggregate in a pattern akin to frost on a branch has been reported in both immunocompetent and immunosuppressed individuals.

Multiplex polymerase chain reaction (mPCR) testing has become an invaluable and elegant tool due to its ability to screen aqueous humor samples for multiple organisms when an infectious uveitic process is suspected. The use of mPCR has been validated in multiple studies, and found to have high sensitivity and specificity [2-6].​ However, as with all laboratory tests, performance metrics depend on a variety of factors. For instance, the primary location of the inflammatory process is likely important. mPCR run on aqueous samples might have a limited ability to detect organisms in a posterior process which. primarily involves the choroid, and retinal vessels with limited involvement and spill over to the vitreous and anterior segment. Situations in which the clinical exam and history are suggestive of an infectious etiology, but a negative mPCR analysis result have been well-described [3]​.​​​​​​ Sample dilution is an additional factor that likely affects performance [4,5]. Although dilution at laboratories is commonly practiced when sample volumes are considered insufficient, the effect on test sensitivity and specificity is unclear. We present a case of FBA in which multiple mPCR tests were negative following sample dilution in direct conflict with the patient presentation, history and clinical course, and discuss how current literature might guide management of these challenging cases.

## Case presentation

A 34-year-old Middle-Eastern female with a past surgical history of three cardiac transplants for hypoplastic left heart syndrome presented to the emergency room in August of 2019 with a two-day history of decreased vision in the left eye. Following the second transplant in 2000, she was diagnosed with and successfully treated for transplant-associated toxoplasmosis. At the time of presentation, her immunosuppressive regimen consisted of cyclosporine 25 mg twice daily, mycophenolic acid 180 mg twice daily, sirolimus 1 mg twice daily, and prednisone 5 mg daily. She was taking no prophylactic antimicrobials. Her past medical history also included hypertension treated with multiple medications, and on admission, her blood pressure was noted to be 165/103. The best corrected visual acuity (BCVa) was 20/20 in the right eye and 20/40 in the left eye. Intraocular pressure was 18mm Hg in both eyes.

**Figure 1:**
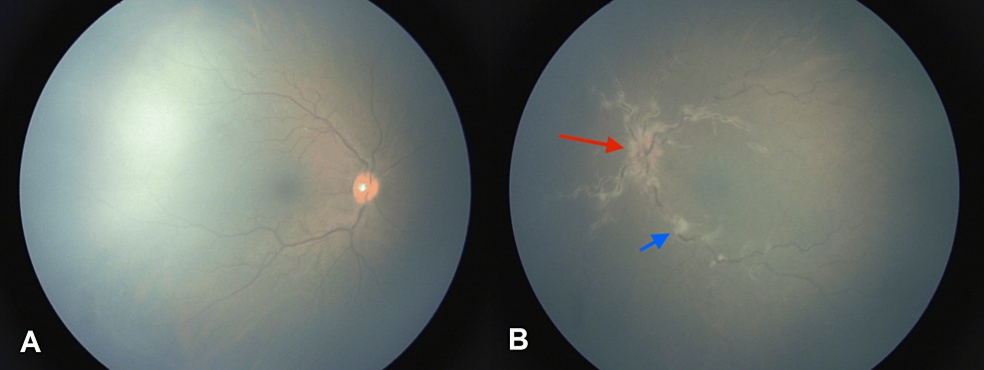
Dilated fundus examination RetCam3 fundus photographs (Natus, San Carlos, CA). A: Grossly normal fundus. B: Frosted branch angiitis, cotton wool spots, and optic nerve edema; red arrow shows optic nerve edema and the short blue arrow shows cotton wool spots

Slit lamp exam of the anterior segment of both eyes was unremarkable, with no cell or flare in the anterior chamber. Dilated fundus exam of the right eye showed moderate vascular tortuosity, while an exam of the left eye presented a rare vitreous cell, peripapillary cotton wool spots (CWS), moderately tortuous vessels, venous sheathing consistent with phlebitis, and dot blot hemorrhages in the periphery (Figure 1).

**Figure 2:**
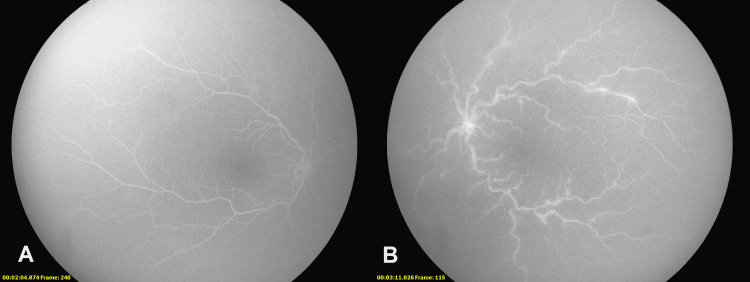
Fluorescein angiography images RetCam3 fundus photographs (Natus, San Carlos, CA). A: Grossly normal fundus. B: Frosted branch angiitis, cotton wool spots, and optic nerve edema.

Fluorescein angiography was obtained revealing normal vascular fill in the right eye and leakage from the optic nerve and venules consistent with papillitis and phlebitis of the left eye without delayed filling (Figure 2).

**Figure 3:**
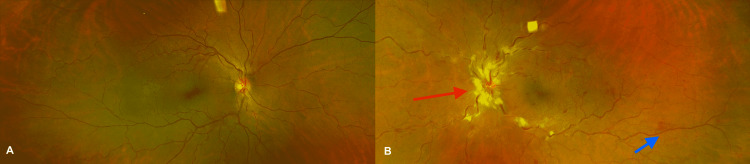
Fundus images at one-week follow-up Optos widefield fundus images (Optos, Marlborough, MA). A: Normal fundus. B: Florid optic nerve edema and cotton wool spots with peripheral intraretinal hemorrhages mostly in the inferotemporal periphery; red arrow: optic nerve edema and blue arrow: peripheral intraretinal hemorrhages

Serologic analysis was positive for *Toxoplasma gondii* IgG 126 IU/ml (normal limit 3-9 IU/ml) with negative IgM. Cytomegalovirus (CMV) IgG was positive >8.0 IU/ml (normal limit 0.2 - 0.8), while IgM was negative. HIV-1 & -2 screening for antigens and antibodies was negative. Herpes simplex virus (HSV) 1 & 2 and CMV serum polymerase chain reaction (PCR) testing were negative, as were Quantiferon Gold and fluorescent treponemal antibody absorption assay (FTA-ABS). Complete blood count with differential was within normal limits. In the setting of an attached vitreous and the concern for inducing a retinal detachment with a vitreous tap, an anterior chamber tap to acquire aqueous humor was elected. A 0.2 cc anterior chamber aqueous sample was obtained and sent to the Mayo Clinic Laboratory for PCR testing, where nucleic acid was extracted by the MagNA Pure automated instrument (Roche Applied Sciences) and amplified by the LightCycler instrument (Roche LifeScience). A negative result was obtained for CMV, *T. gondii*, HSV-1 & -2, varicella-zoster virus (VZV), and Epstein-Barr virus (EBV), though the sample was diluted at the laboratory prior to processing due to insufficient volume of the original specimen. MRI of the brain and orbits with and without contrast revealed scattered, remote, and bilateral cerebral and cerebellar micro-hemorrhages consistent with a history of heart transplantation due to micro-emboli and transitory hypoxic injury. Hospital admission was elected for infectious disease and transplant medicine consultation, as well as to optimize follow-up and proper intravenous and intravitreal antiviral treatment. During admission, the patient received intravenous ganciclovir 5 mg/kg IV (intravenous) twice a day, and trimethoprim/sulfamethoxazole (TMP/SMX) 160/800 mg tablets were given twice daily. During her hospital course, her vision improved to 20/25 in the left eye. Blood pressure normalized and was stable at 113/70 on discharge, at which point intravenous ganciclovir was switched to oral (PO) valganciclovir 900 mg, and TMP/SMX was continued at the same dose. The patient continued with immunomodulatory medications to avoid organ transplant rejection including prednisone 15 mg PO daily. On one-week follow-up BCVa was stable in the left eye. There was an improvement in phlebitis on the fundus examination (Figure 3).

**Figure 4:**
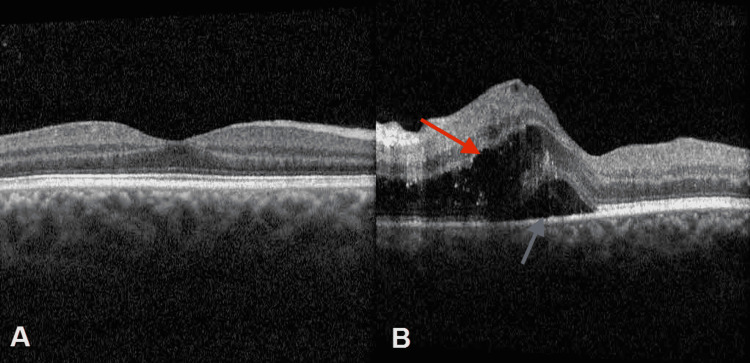
Optical coherence tomography images Heidelberg optical coherence tomography (Heidelberg Engineering, Heidelberg, Germany). A: Optical coherence tomography of the right eye with intraretinal cysts and edema after decreasing dosage of oral valganciclovir in the photoreceptor layer; B: The red arrow shows intraretinal cysts and the gray arrow shows subretinal fluid

Intraretinal and subretinal fluid was observed on Optical Coherence Tomography (OCT) (Figure 4).

**Figure 5:**
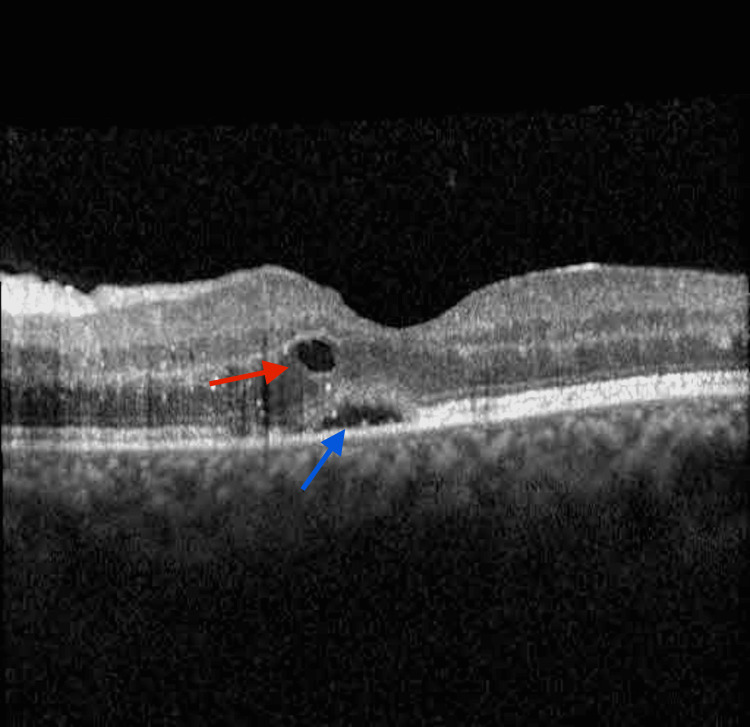
Optical coherence tomography image of the left eye demonstrating improvement in thickness and edema after one week Red arrow: intraretinal cysts; blue arrow: subretinal fluid

Continued improvement was seen on the exam one week later as evidenced by the reduction in macular edema (Figure 5).

**Figure 6:**
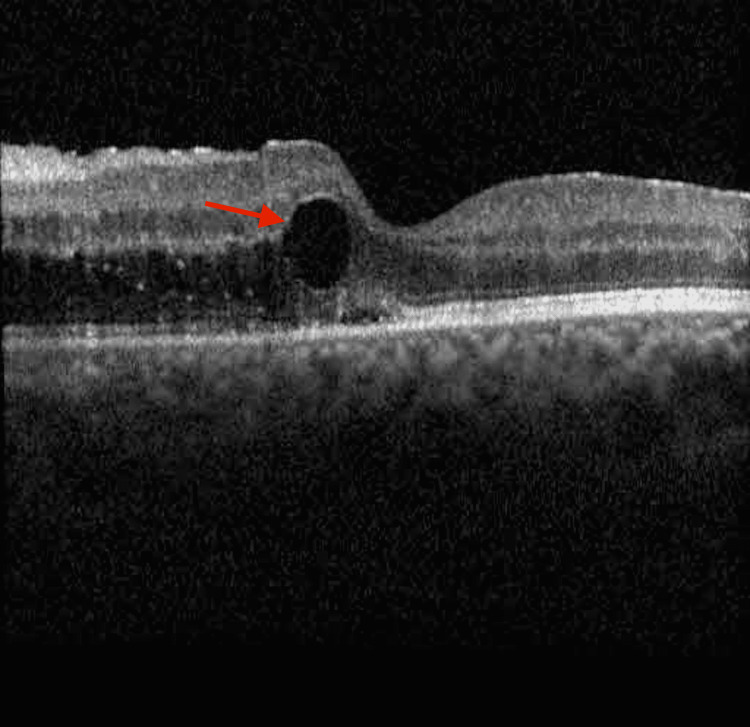
Optical coherence tomography demonstrating the worsening of intraretinal cysts and edema Red arrow: intraretinal cyst

The valganciclovir dose was reduced to 450 mg orally twice a day. One week later, the vision had deteriorated to 20/50, and OCT demonstrated a worsening of the intraretinal fluid (Figure 6).

**Figure 7:**
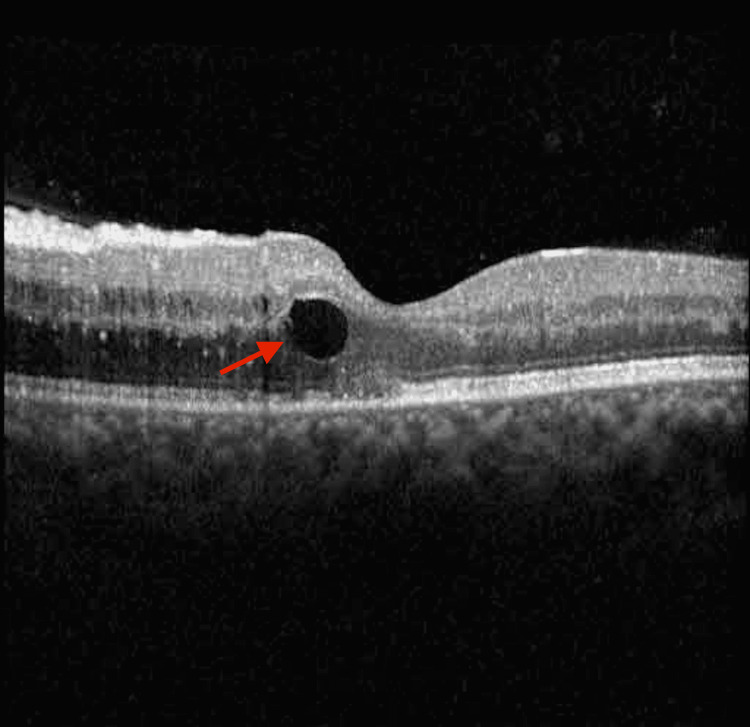
Optical coherence tomography demonstrating mild improvement in thickness one week after discharge Red arrow: intraretinal cysts

Valganciclovir was increased to 900 mg twice a day, and a lumbar puncture was performed with cerebrospinal fluid (CSF) analysis including a PCR panel for *T. gondii*, EBV, CMV, enterovirus, HSV-1 & -2, HHV-6, human parechovirus (HPeV), VZV, *Cryptococcus neoformans*, *Cryptococcus gattii*, *Eschericia coli* K1, *Haemophilus influenzae*, *Listeria monocytogenes*, *Neisseria meningitidis*, Group B Streptococcus, and *Streptococcus pneumoniae*, as well as culture, Gram stain, fungal stain, oligoclonal banding, flow cytometry for CD19, lambda and kappa and CD45 markers. The entirety of the CSF analysis was negative. Anterior chamber paracentesis was repeated and a 0.25 cc sample was sent as before for PCR of HSV-1 & -2, VZV, CMV, EBV, and *T. gondii*, and concurrent intravitreal injection of ganciclovir 2 mg/0.05 cc was given. This second sample was, again, diluted at the laboratory prior to processing due to insufficient volume of the original specimen, and was read as negative. OCT appearance demonstrated mild improvement one week after discharge (Figure 7).

**Figure 8:**
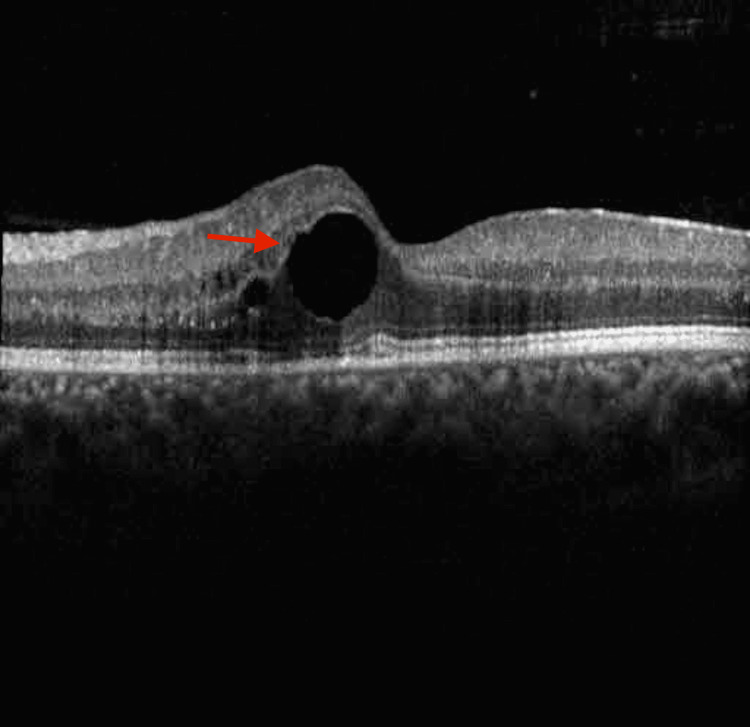
Optical coherence tomography with worsening of intraretinal cysts without subretinal fluid Red arrow: intraretinal cysts, peripheral micro-cysts

On exam one week later, improvement of the peripapillary CWS was noted, but there was an increase in the amount of dot blot hemorrhages in the periphery. Coupled with worsening intraretinal edema (Figure 8). These findings were suggestive of non-ischemic central retinal vein occlusion (CRVO) with moderate optic nerve edema, given that the patient's vision was better than 20/200, and the absence of an afferent pupillary defect. Non-ischemic CRVO was confirmed with less than ten areas of non-perfusion on fluorescein angiography.

**Figure 9:**
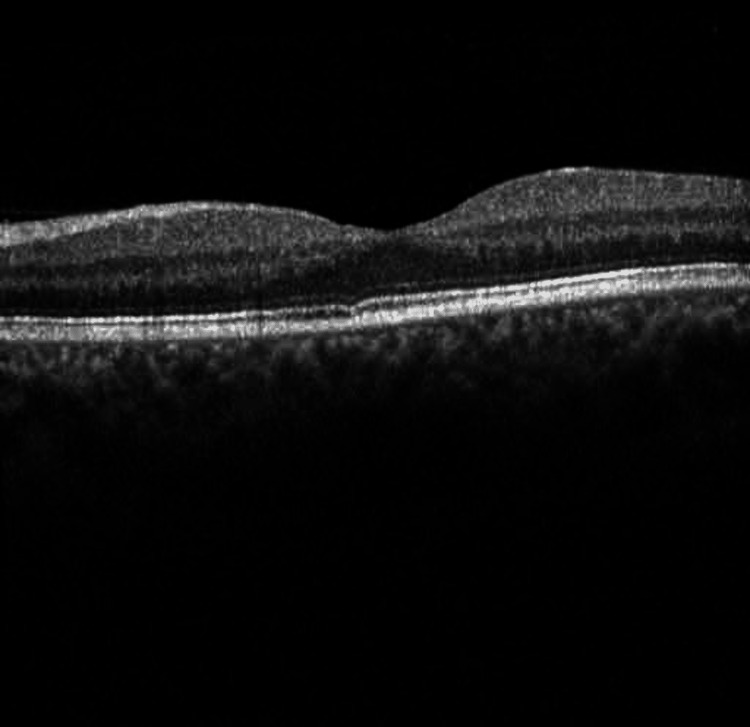
Optical coherence tomography of the left eye after intravitreal bevacizumab administration

Intravitreal injection of bevacizumab was given. One week later, BCVa improved to 20/25 OS (left eye) and there was an anatomic improvement on OCT (Figure 9).

**Figure 10:**
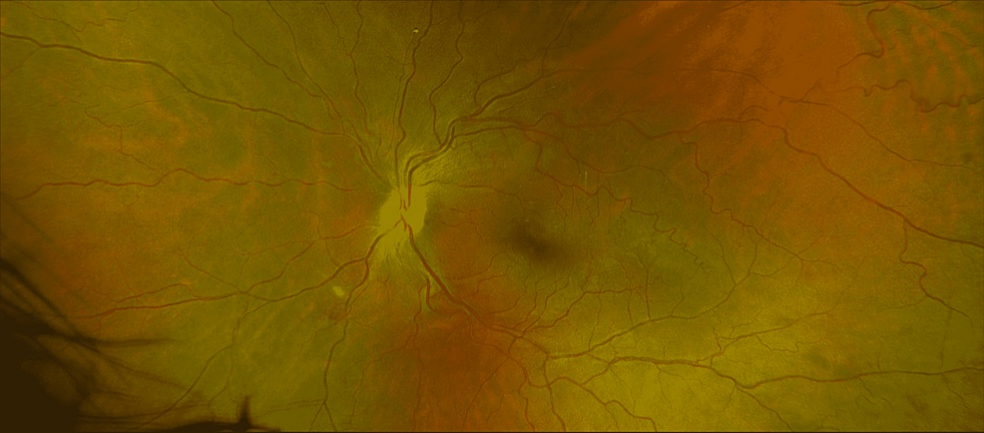
Fundus photograph of last follow-up appointment at 6 months from initial presentation Fundus photograph of last follow-up appointment at 6 months from initial presentation demonstrating improvement and near total resolution of cotton wool spots and hemorrhages

One month after bevacizumab injection the intraretinal cysts had completely resolved, and the valganciclovir dose was again decreased to 450 mg BID (twice a day) followed by discontinuation of TMP/SMX. At 6 months from presentation, there was almost complete resolution of the peripapillary CWS and peripheral hemorrhages of the left eye, final BCVa of 20/20 OS (Figure 10).

## Discussion

FBA has a striking fundus appearance; the retinal vasculitis is widespread and may be bilateral with a ‘frosted’ quality to the perivascular exudate. Mild-to-moderate iritis with vitritis is common, as is intraretinal edema [7]. It has been postulated that this vasculitis is an idiosyncratic type 3 immune-complex response to an offending antigen. When the etiology is infectious, CMV is often implicated [7]. ​​​​​​ In a review by Walker et al., the majority of cases were determined to be non-infectious and were accordingly treated with steroid therapy [6]. The mainstay of treatment in infectious cases is systemic antiviral therapy. Intravitreal injections of antiviral medications are used as an adjunctive strategy in cases of rapidly progressive disease [7].

In the past, the process of identifying the causative pathogen in cases of infectious uveitis depended on acquiring viral cultures or on the Goldmann-Witmer coefficient. The development of monoplex PCR and eventually the validation of multiplex PCR analysis created a pivotal laboratory tool in the management of infectious uveitis. Multiplex PCR introduced the ability to combine different primer pairs in the same amplification reaction, allowing one series of amplifications to produce multiple specific infectious amplicons from a specimen of intraocular fluid. In our case, the lack of vitritis or retinitis likely limited the sensitivity of PCR, since scant shedding of infectious particles may be insufficient to yield a positive result [8,9]. ^ ^In a study by Nakano et al., it was reported that the patient cohort with robust panuveitis had higher viral loads than patients presenting with anterior uveitis alone [2]. In this series, a patient with frosted branch angiitis of presumed infectious etiology had a negative PCR result. Interestingly, other select cases of presumed infectious etiology such as neuroretinitis and cat scratch disease were PCR negative [2]. In contrast, an article by Harper et al. identified factors positively correlated with successful mPCR diagnosis, including immunocompromised status i.e., HIV+ (given larger retinal lesion extension), optic nerve involvement, and visible retinal vascular lesions [5].

It is important to note that the number of copies needed to trigger a positive result varies between laboratories. To date, there is no standardized reporting method or nucleic acid concentration (i.e. copies/ml) used to establish universal PCR results. Moreover, within laboratories, different detection limits exist between organisms. For example, Haper et al. discuss that at the lab used in their methods section, the detection limit for the herpes simplex viral test was 100 copies/ml, while the detection limits in the Chichili et al. paper the detection limit for HSV-1 was expressed as 4 PFU/ml which corresponds to 20 copies/ml [2-5,8]. ^ ^

In an article put forth by Schoenberger and collaborators, up to 21% of cases with presumed herpetic retinitis had negative PCR testing [9]. Some studies estimate the rate of a false-negative PCR result when faced with a compelling clinical picture and diagnosis of viral retinitis is around 14% [9-11].^ ^In regards to similar infectious processes, in a study by Elyashiv et al., 42% of eyes with PCR-positive herpetic ocular disease had atypical presenting signs such as optic nerve head edema or vasculitis [12]. Although these data should be interpreted with caution as the study was based on photographic analysis only, it may mean that some 58% of PCR-proven cases of herpetic retinitis would not have qualified in the criteria set forth by the American Uveitis Society, creating a discordant image between the clinical appearance and PCR analysis [9].​​​​​​

Solid organ transplantation has become a risk factor given the high doses of steroids used to avoid acute rejection as well as the chronic use of immunomodulatory therapy to increase graft survival. Ng et al. retrospectively evaluated 860 patients after solid organ transplantation and documented a variety of ocular features in 19 uveitic patients [13]. ​​​​​​ An infectious process was diagnosed in 65% of these patients, with the herpes virus being the causative agent in 88% of cases. The second year post-transplantation was the most common time of onset for ocular complications [11-15]. In another review by Quinlan et al., in regards to ocular complications after cardiac transplantation, two patients (3.4%) out of 59 developed cytomegalovirus retinitis which presented within 6 months of the transplantation [15].^ ^ It is important to consider that immunodeficient patients do not present exuberant inflammation in the setting of a viral retinal and necrotic process, given the blunted immune response. This is often found in patients suffering from acquired immunodeficiency syndrome, as well as in patients treated with multiple immunomodulatory agents.

In summary, multiple authors have demonstrated that multiplex PCR has a high specificity rate, but a dilute sample could potentially lower the yield and sensitivity. Even in this setting, and even with serial dilution, the test maintains excellent sensitivity and specificity. Despite the high reliability of PCR in most instances, a primarily posterior inflammatory process without significant anterior segment cellularity may be likely to give false-negative results, such as in the case of FBA detailed here. Clinical examination and close follow-up remain crucial to evaluate the worsening of the disease in cases of delayed treatment given the diagnostic uncertainty in the setting of a negative aqueous sample. In cases where an infectious etiology is highly suspected the in-patient setting is ideal, as it provides the advantages of rapid involvement of a multidisciplinary team and allows for faster diagnosis and adjustments in treatment. 

## Conclusions

We believe that the protean nature of the posterior infectious uveitis merits careful consideration, therefore the therapeutic algorithm should be crafted on an individual basis. Multiplex PCR has excellent sensitivity and specificity, even in the setting of a dilute sample. False-negative results are possible but rare. Empiric systemic treatment in patients with presumed infectious posterior uveitis is the best treatment course. In particular, this case demonstrates the challenge of establishing a clear diagnosis in a case of frosted branch angiitis in a patient post cardiac transplantation on chronic immunosuppression.
